# A Bifunctional Fluorescence Probe for the Detection of Hypochlorous Acid and Viscosity in Living Cells and Zebrafish

**DOI:** 10.3390/molecules30071531

**Published:** 2025-03-30

**Authors:** Xin Zhang, Yanmei Si, Xinpeng Chen, Xuqing Nie, Yiheng Zhang, Li Lin, Yehao Yan

**Affiliations:** 1 School of Public Health, Shandong Second Medical University, Weifang 261053, China; zhangx_0928@163.com; 2 School of Public Health, Jining Medical University, Jining 272067, China; n2796982732@163.com (X.N.); z19558669058@163.com (Y.Z.); 3 School of Forensic Medicine and Laboratory Medicine, Jining Medical University, Jining 272067, China; siyanmei90@126.com; 4 School of Life Science, Hubei Normal University, Huangshi 435002, China; chenxinpeng@hbnu.edu.cn

**Keywords:** fluorescence probe, FRET, hypochlorite, viscosity

## Abstract

As two significant indicators in the microenvironment, hypochlorous acid and viscosity play important roles in multitudinous physiological metabolic processes. However, it is challenging to determine the dynamic levels of hypochlorous acid and viscosity in living cells and organisms because of the absence of effective molecular tools that can simultaneously detect hypochlorous acid and viscosity in organisms. Herein, a molecular design strategy was presented to fabricate a single fluorescence probe that can simultaneously detect hypochlorous acid and viscosity by using two different emission channels. In JXR, TICT-based 4-(2-(5-(dimethylamino)thiophen-2-yl)vinyl)-1-methylpyridin-1-ium-iodide chromophore serves as energy acceptor in the FRET process and sensors for hypochlorous acid and viscosity. JXR showed a large Stokes shift, wide emission peak distance, high photostability, and low toxicity. JXR could detect hypochlorous acid and viscosity rapidly, sensitively, and selectively by emitting different fluorescence signals. Importantly, JXR was successfully applied to track the intracellular hypochlorous acid and viscosity in living cells. Meanwhile, the generation of endogenous hypochlorite in living cells can be observed by using JXR.

## 1. Introduction

As an important oxidizing species in organisms, hypochlorous acid (HClO) is commonly produced in neutrophils and leukocytes [1,2] and plays a significant role in various physiological metabolic processes including apoptosis regulation, inflammation factors inhibition, and immune defenses [3,4,5]. Unregulated HClO concentration in organisms is generally associated with some disorders, such as atherosclerosis, rheumatoid arthritis, and even cancer [6,7,8]. In addition, viscosity is another essential parameter in the microenvironment [9,10], which is involved in many biological processes in vivo, such as membrane fusion, signal transduction, cellular metabolism, and migration [11,12,13]. Abnormal viscosity increase is closely associated with some diseases including cardiovascular diseases, diabetes, and tumors [14,15,16]. Nevertheless, the non-invasive, real-time, and in situ detection is still a challenge for HClO and viscosity in living organisms.

Compared with traditional detection techniques, fluorescence probes were developed rapidly and considered an effective detection approach for biochemical analysis due to the superiorities of prominent biocompatibility, real-time detection, low detection limit, high selectivity, and sensitivity [17,18,19,20]. Presently, fluorescence probes for the individual detection of HClO and viscosity have been massively developed [21,22,23]. However, the progress of the ratiometric fluorescence probe is still limited for the simultaneous detection of HClO and viscosity [24,25,26,27]. Ratiometric fluorescence probes commonly exhibit high accuracy by plotting the ratio of dual emission intensity and effectively eliminating the disturbances from environments, instruments, and manipulations [28,29]. The common molecular building mechanisms of fluorescence probe are intramolecular charge transfer (ICT) [30,31], photoinduced electron transfer (PET) [32,33], FÖrster resonance energy transfer (FRET) [34,35], excited state intramolecular proton transfer (ESIPT) [36,37], and through-bond energy transfer (TBET) [38,39]. Compared with other mechanisms, the FRET platform can be easily used to design ratiometric fluorescence probes composed of energy donors, linkers, and energy acceptors [40,41]. Furthermore, the twisted intramolecular charge transfer (TICT) mechanism has been utilized to construct viscosity-sensitive fluorescence probes [42,43]. Although some FRET/TICT-based probes for SO_2_ derivatives and viscosity were developed, HClO and viscosity dual response probes based on the FRET/TICT platform were less (Table S1).

**Scheme 1:**
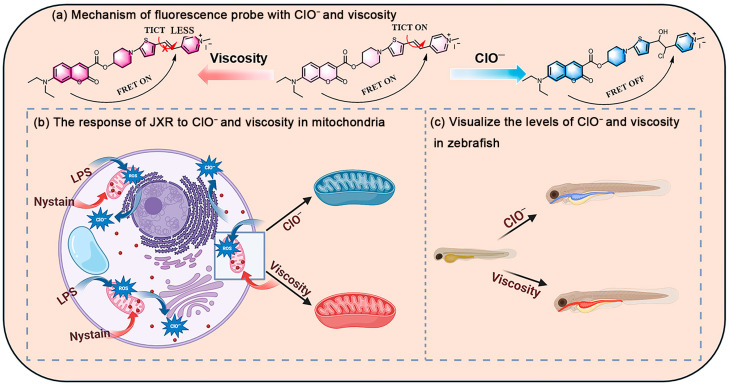
Probe JXR detects HClO and viscosity in living cells and zebrafish.

Herein, a novel dual response fluorescence probe JXR was prepared to detect HClO and viscosity based on the FRET and TICT platforms. The normalized emission spectra of the donor overlapped well with the normalized absorbance spectra of the acceptor, which provided a high energy transfer efficiency of 82.5% (Equation (S1)) (Figures S1 and S2). The absolute fluorescence quantum yield of JXR was measured to be 0.6%. The molar extinction coefficient is 2.5 × 10^4^ L/mol/cm [44]. JXR possesses a high energy transfer efficiency (82.5%), large Stokes shift (194 nm), and wide emission spacing (124 nm). With the continuous addition of HClO, JXR emitted a gradually quenched red emission signal at 594 nm and a sustainably strengthened blue emission signal at 470 nm, which is the foundation of ratio detection (Scheme 1). Additionally, the increasing viscosity of the testing system effectively suppressed the intramolecular rotation of the acceptor moiety, and the red emission signal at 594 nm was gradually strengthened to detect viscosity. Importantly, JXR has been successfully applied to detect the varying levels of HClO and viscosity in living cells and zebrafish. Consequently, a bifunctional fluorescence probe for HClO and viscosity in living cells and in vivo was successfully developed.

## 2. Result and Discussion

### 2.1. Response Properties

**Figure 1:**
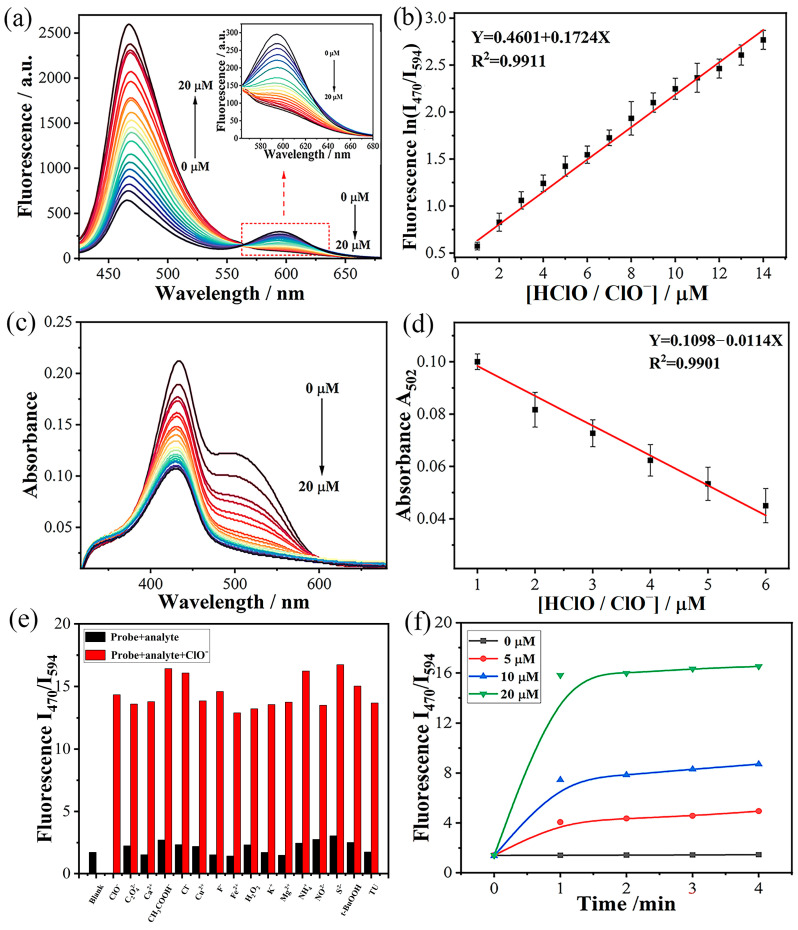
(**a**) The fluorescent spectrum of JXR (5 μM) toward HClO (0–20 μM). (**b**) The linearity between ln (I_470_/I_594_) and HClO concentrations. (**c**) The absorbance spectrum of JXR for HClO (0–20 μM). (**d**) The linearity between A_502_ and HClO concentrations. (**e**) The fluorescence intensity ratio (I_470_/I_594_) of JXR (5 μM) toward various analytes (20 μM). (**f**) The response time of JXR versus HClO.

The response behaviors of JXR toward HClO and viscosity were thoroughly assessed. Probe JXR displayed two emission peaks at 470 and 594 nm under the excitation of 400 nm. Upon the increase in HClO concentration in the testing system, the blue fluorescence signal at 470 nm intensified gradually and the red fluorescence signal at 594 nm abated unceasingly (Figure 1a). The above varying can be explained to be the reaction between the acceptor moiety and HClO causing the cessation of the FRET and TICT platform [45,46]. Naturally, an excellent linear relationship of y = 0.4601 + 0.1724x with R^2^ = 0.9911 was presented by plotting the increase in I_470_/I_594_ depending on the increase in HClO concentration (Figure 1b). Simultaneously, the detection limit of JXR toward HClO was calculated as 95.7 nM based on LOD = 3σ/K where σ was 0.0055 and K was 0.1724 (Equation (S2)). In UV-vis spectra, the absorption signal located at 502 nm is reduced with the continuous increase in HClO contents in the testing system, which is consistent with the differences in fluorescence spectra (Figure 1c,d).

Furthermore, the specificity and anti-interference capacity of JXR for HClO was researched. Initially, the effect of many species was estimated, such as blank, HClO, C_2_O_4_^2−^, Ca^2+^, CH_3_COO^−^, Cl^−^, Cu^2+^, F^−^, Fe^2+^, H_2_O_2_, K^+^, Mg^2+^, NH_4_^+^, NO_2_^−^, S^2−^, t-BuOOH, and Thiourea (TU) (Figure 1e). Compared with the blank group, the fluorescence intensity ratio (I_470_/I_594_) of JXR changed obviously in the presence of HClO, while it exhibited negligible discrepancies in the presence of other interfering species. In addition, JXR could respond well with HClO although many interfering species were presented in the testing system. These experimental results suggested that probe JXR with higher specificity for HClO than other competitive interfering species and prominent anti-interference capacity in the presence of other species.

Next, the response time of JXR toward HClO was determined by plotting the response kinetic profile between the I_470_/I_594_ and reaction time. In only the JXR-existing system, the ratio of I_470_/I_594_ no obvious varying could be observed, suggesting the excellent photostability in solution. After HClO was added to the testing system, the ratio of I_470_/I_594_ increased rapidly and raised to a maximum of about 1 min (Figure 1f), which effectively demonstrated the rapid response feature of JXR toward HClO. In addition, the pH effects of JXR on the fluorescence and response properties were determined. The fluorescence intensity ratio (I_470_/I_594_) of JXR remains invariable in an extensive pH range (Figure S3a). After adding HClO to the testing system, JXR could react well with HClO at a pH range between 5.0 and 10.0. Meanwhile, in the fluorescence and absorbance spectra of JXR, no clear changes could be observed (Figure S3b,c). These testing results revealed that JXR possesses a favorable pH stability and a good response capacity for HClO in organism conditions. Then, the solvent effects of JXR were measured in different organic solvents including dichloromethane, 1,4-dioxane, tetrahydrofuran, N,N-dimethylformamide, and dimethyl sulfoxide. The emission and absorbance spectra of JXR were changed obviously in different solvents (Figure S3d,e).

**Figure 2:**
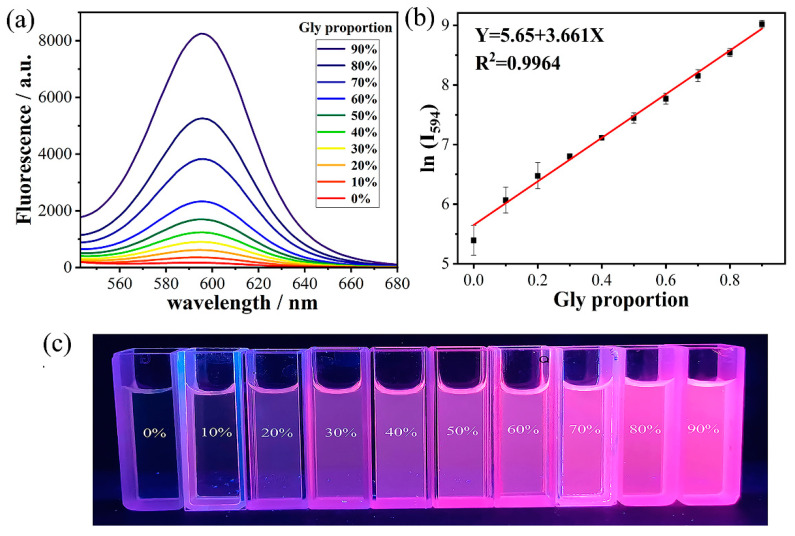
(**a**) The fluorescence spectra of JXR (5 µM) in different ratios of PBS-glycerol mixtures. (**b**) The linear correlation between ln I_594_ and glycerol proportion. (**c**) The variation in fluorescence of JXR in different ratios of a PBS-glycerol mixture under ultraviolet light.

Furthermore, the response of JXR toward viscosity was also appraised in testing solutions. The viscosity of the experimental system was enhanced continuously by increasing the glycerol contents in the glycerol/PBS solution [47,48]. In a pure PBS (phosphate-buffered saline) testing system, JXR emitted a negligible fluorescence at 594 nm since the presence of the TICT effect in acceptor moiety [49,50] (Figure 2a). The increasing viscosity in the testing system effectively alleviated the TICT effect and led to a gradually strengthened fluorescence at 594 nm. Compared with the PBS system, the fluorescent intensity of JXR strengthened more than 30-fold in glycerol/PBS mixtures. Meanwhile, the fluorescence intensity at 594 nm showed a preferable linearity with the increasing glycerol proportion (R^2^ = 0.9964, y = 3.661x + 5.65) (Figure 2b). In addition, JXR emitted a continuously enhanced red fluorescence under 365 nm ultraviolet light with the increasing viscosity of the testing system (Figure 2c). The above outcomes suggested that JXR with the potential to detect viscosity.

### 2.2. Fluorescence Imaging in Living Cells

**Figure 3:**
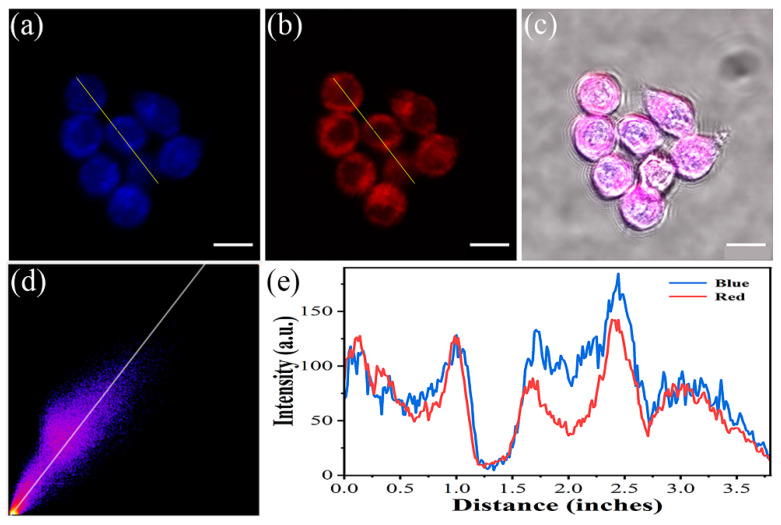
(**a**) Confocal fluorescence imaging for RAW264.7 cells stained with JXR (2 μM). (**b**) Imaging for RAW264.7 cells stained with Mito-Tracker Deep Red dyes (200 nM). (**c**) Overlapped imaging. (**d**) Colocalization imaging (0.95). (**e**) Fluorescent line intensity for the valuation of the overlapping of Mito-Tracker Deep Red dyes (red line) with JXR (blue line). Scale bars: 10 μm.

Based on the prominent fluorescence properties of JXR, the potential to trace intracellular HClO and viscosity levels was further researched in RAW264.7 cells. Firstly, the biocompatibility of JXR toward living cells was assessed through CCK8 assay in RAW264.7 cells [51,52]. The survival rate of these cells with almost no notable reduction was detected even when the concentration was as high as 5 μM, illustrating the good biocompatibility of JXR in living cells (Figure S4). Secondly, the photostability of JXR in living cells was also estimated. JXR in RAW264.7 cells exhibited a stable fluorescence signal under continuous light exposure, which demonstrates the high photostability of JXR in cells (Figure S5). Then, the organelle-targeted feature of JXR was also explored by co-staining RAW264.7 cells with JXR and Mito-Tracker Deep Red dyes [53,54]. The blue emission signal of JXR almost overlapped completely with the red emission signal of Mito-Tracker Deep Red dyes with a favorable colocalization coefficient of 0.95, which suggested the specific mitochondria-targeted capacity of JXR in cells (Figure 3). The feature was generated from the electrostatic interaction originating from the cation moiety of JXR with the negative charge in the mitochondria membrane [55,56]. The mitochondria-targeted ability was better than other HClO and viscosity dual response probes (Table S2).

**Figure 4:**
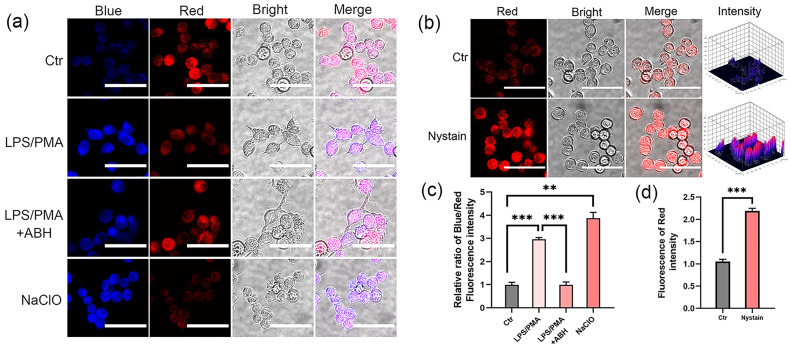
The confocal fluorescence imaging of JXR in living RAW264.7 cells. (**a**) First line: cells were cultured for 0.5 h with 2 μM JXR. Second line: after cultivation for 12 h with LPS (2 μg/mL) and PMA (1 μg/mL), cells were cultured for 0.5 h with 2 μM JXR. Third line: after cultivation for 12 h with LPS/PMA, cells were cultured for 1 h with ABH (1 μg/mL), and then cultured for 0.5 h with 2 μM JXR. Fourth line: after cultivation for 1 h with 100 μM NaClO, cells were cultured for 0.5 h with 2 μM JXR. (**b**) First line: cells were cultured for 0.5 h with 2 μM JXR. Second line: after cultivation for 2 h with 50 μM nystatin, cells were cultured for 0.5 h with 2 μM JXR. (**c**) The relative ratio of I_blue_/I_red_ of imaging in (**a**). (**d**) The relative fluorescence intensity of imaging in (**b**). The imaging experiment was repeated three times (*n* = 3), and the results were exhibited as mean ± standard deviation, ** *p* < 0.01, *** *p* < 0.001. Scale bars: 50 μm.

Next, the responding feature of JXR toward endogenous HClO was also determined in RAW264.7 cells. The cells in the control group only incubated with JXR could emit weak signals in the blue window and intense signals in the red window (Figure 4a). However, these cells initially hatched with Lipopolysaccharide/Phorbol-12-myristate-13-acetate (LPS/PMA) and subsequently hatched with JXR could emit a weakened signal in the red window and intensified signal in the blue window, which was caused by the production of endogenous HClO in living RAW264.7 cells under the catalysis of LPS/PMA [57]. 4-Aminobenzohydrazide (ABH) is a myeloperoxidase inhibitor and commonly used to inhibit hypochlorite production [47]. In the presence of ABH, these cells hatched with LPS/PMA and JXR still without obvious variations could be observed compared with the control group. Meanwhile, the performance of JXR response to exogenous HClO was also evaluated. These cells were initially hatched with JXR and followed by treatment with NaClO before imaging. The results are presented in Figure 4a; the testing cells could emit a strengthened signal in the blue window and a weakened signal in the red window compared to the control group. Furthermore, the relative fluorescence intensity ratio value (blue/red) of the above cells exhibited consistent changes with images (Figure 4c). Therefore, probe JXR could effectively detect endogenous and exogenous HClO in living RAW264.7 cells.

Moreover, the responding feature of probe JXR toward intracellular viscosity was also researched. The intracellular viscosity could be regulated by hatching the experimental cells with nystatin [58,59]. In the control group, these cells exhibited a very weak signal in the red window after the hatching of JXR alone (Figure 4b). In the experimental group, before hatched with JXR, these cells were initially hatched with nystatin. The signal in the red window was intensified markedly, and the relative fluorescence intensity was increased obviously (Figure 4d). These results clearly revealed that JXR could successfully detect the varying intracellular viscosity and possessed a prominent potential in the diagnosis of viscosity-related illnesses.

### 2.3. Bioimaging in Zebrafish

**Figure 5:**
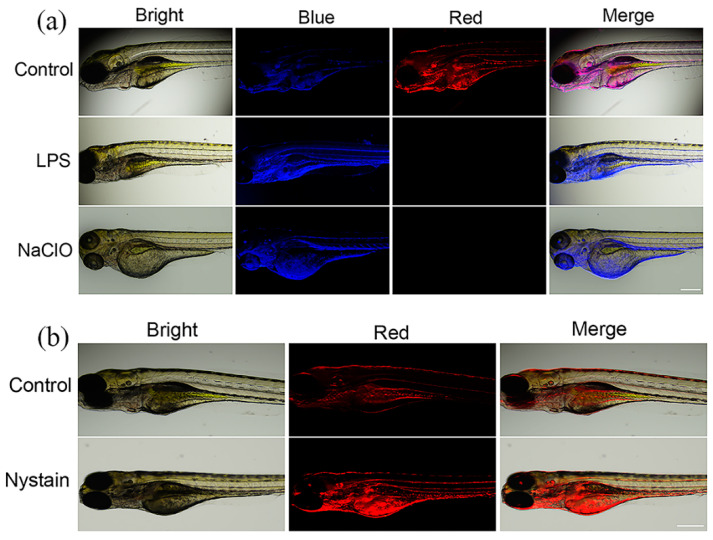
The bioimaging of probe JXR in living zebrafish model. (**a**) First line: zebrafish cultured for 0.5 h with JXR (4 μM). Second line: zebrafish cultured for 2 h with LPS (5 μg/mL), and then cultured for 0.5 h with JXR (4 μM). Third line: zebrafish cultured for 1 h with NaClO (10 μM), and then cultured for 0.5 h with JXR (4 μM). (**b**) First line: zebrafish cultured for 0.5 h with JXR (4 μM). Second line: zebrafish cultured for 2 h with nystatin (50 μM), and then cultured for another 0.5 h with JXR (2 μM). Scale bars: 200 μm.

Encouraged by the prominent properties of JXR in living cells, the viability of JXR to detect HClO and viscosity in vivo was further researched. As a common vertebrate model in fluorescence imaging, zebrafish was selected to fulfill this experiment, and the result is shown in Figure 5a. The zebrafish hatched with JXR could emit a weak signal in the blue window and an intense signal in the red window. After the pre-incubation with NaClO, the JXR-incubated zebrafish could emit a strengthened signal in the blue window and a weakened signal in the red window. Meanwhile, after the pre-incubating of LPS, JXR-incubated zebrafish also presented a congruous varying with the above result. In addition, nystatin was usually used to hatch zebrafish to enhance the viscosity of their living organisms. In the control group, zebrafish were incubated only with JXR and emitted a feeble signal in the red window (Figure 5b). However, the zebrafish first incubated with nystatin and subsequently incubated with JXR could emit a strengthened signal in the red window compared with the control group. The above variations were consistent well with the varying in living cells and indicated that JXR has a great potential for the visualization of HClO and viscosity in vivo.

## 3. Experiments

### 3.1. Reagents and Instruments

The experimental reagents were purchased from commercial providers and utilized directly. Bruker Avance 400 MHz spectrometer (TMS, DMSO-*d*_6_, Bruker, Billerica, MA, USA) was utilized to obtain ^1^H NMR and ^13^C NMR spectra. Agilent Q-TOF6510 spectrometer was utilized to obtain HR-MS (Agilent Technologies, Santa Clara, CA, USA). Hitachi F-2700 fluorometer (Hitachi, Tokyo, Japan) was utilized to measure the fluorescence signal (Excitation wavelength: 400 nm, Slit: 10/10 nm, Scan speed: 300 nm/min, Voltage: 700 V). TU-1901 spectrophotometer was utilized to measure UV-vis absorbance spectra. Zeiss laser confocal microscope (Zeiss, Jena, Germany) was utilized to conduct cell imaging experiments (Excitation wavelength: 405 nm, blue channel: 430–550 nm, red channel: 560–650 nm). PHS-3C pH-meter (LABO-HUB, Shanghai, China) was utilized to determine the pH value of testing solutions. The absolute quantum yield of the sensor was measured by Edinburgh Instruments FLS920 Fluorescence Spectrometer in PBS testing solution (Edinburgh Instruments, Livingston, UK).

Macrophages (Raw 264.7) were procured from Zishan Biotechnology (Servicebio, Wuhan, China). And, the zebrafish experiment was reviewed and approved on 22 September 2024 by the Ethics Review Committee of Jining Medical University, with the code number JNMC-2025-DW-020.

### 3.2. Spectral Measurements and Cell Imaging

The spectral measurements were determined in PBS buffer containing 0.5% DMSO with pH 7.4 at room temperature. The JXR stock solution with 10^−3^ mol/L was obtained by dissolving in DMSO solvent. The various analytical species in experiments were prepared based on previous reports [60,61], and then added to the testing solutions and tested the emission and absorption spectra, respectively.

The RAW264.7 cells were cultured in a DMEM medium containing 1% antibiotics and 10% FBS. The cells were cultured in 96-well plates in a moist 5% CO_2_ atmosphere at 37 °C. The cytotoxicity of this probe to cells was evaluated in living RAW264.7 cells by using standard CCK8 methods. Hatching of 1-phenyl-2-thiourea (PTU)-pretreated zebrafish embryos in E3 medium continued for 3–5 days at room temperature. Zebrafish imaging was obtained by a Nikon ECLIPSE Ti2-U inverted fluorescence microscope (Nikon, Tokyo, Japan).

### 3.3. Synthesis of JXR

**Scheme 2:**
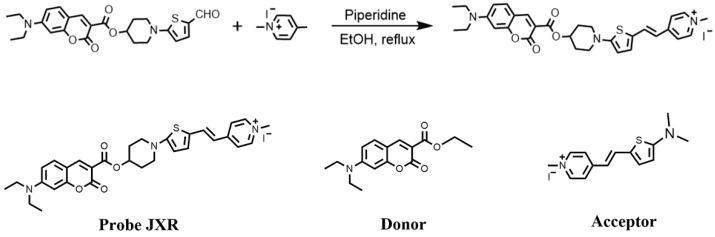
The synthesis of probe JXR.

1-(5-formylthiophen-2-yl)piperidin-4-yl 7-(diethylamino)-2-oxo-2H-chromene-3-carboxylate (0.5 mmol, 227 mg) and 1,4-dimethylpyridin-1-ium-iodide (0.6 mmol, 141 mg) were mixed in anhydrous ethanol (Scheme 2). After refluxing for 12 h under the catalysis of piperidine, the reaction solution was cooled to room temperature. After removing the solvents, further purification was performed by recrystallizing assays in ethanol, and then the resulting compound with 36% yield was obtained. The resulting compound was determined by ^1^H NMR and ^13^C NMR (Figures S6–S8). HR-MS (ESI, *m*/*z*): [C_31_H_34_N_3_O_4_S]^+^: 544.2265, found: 544.2285. ^1^H NMR (400 MHz, DMSO-*d_6_*): δ = 1.14 (t, *J* = 6.0 Hz, 6H), 1.23 (s, 4H), 3.47–3.63 (m, 8H), 4.11 (s, 3H), 5.15 (s, 1H), 6.35 (s, 1H), 6.52 (d, *J* = 13.2 Hz, 2H), 6.79 (d, *J* = 8 Hz, 1H), 7.29 (s, 1H), 7.65 (d, *J* = 8.0 Hz, 1H), 7.90 (d, *J* = 6.0 Hz, 2H), 8.07 (d, *J* = 16.0 Hz, 1H), 8.56 (d, *J* = 6.0 Hz, 2H), 8.63 (s, 1H). ^13^C NMR (101 MHz, DMSO-*d*_6_): 12.8, 29.5, 44.8,47.2, 68.7, 96.3, 105.6, 107.5, 107.7, 111.3, 121.6, 125.1, 126.6, 128.5, 132.3, 141.9, 144.3, 145.0, 149.9, 153.4, 157.5, 158.6, 163.2, 167.6, and 180.7.

## 4. Conclusions

Herein, a single fluorescence probe that can detect hypochlorite and viscosity synchronously by using two different emission channels was designed and synthesized based on the FRET/TICT mechanism. JXR showed a large Stokes shift, wide emission peak distance, and low toxicity. JXR could detect hypochlorite levels, sensitively, and selectively rapidly by calculating the ratio of the dual fluorescence channel intensity. Meanwhile, JXR could also monitor the viscosity level by plotting the linearity between the fluorescence intensity in the red channel and the viscosity in the testing system. Importantly, JXR was successfully applied to track the intracellular hypochlorite and viscosity levels in living cells and zebrafish. Meanwhile, the generation of endogenous hypochlorite in living cells can be observed by using JXR.

## Data Availability

The data presented in this study are available upon request from the corresponding author.

## Supplementary Materials

The following supporting information can be downloaded at https://www.mdpi.com/article/10.3390/molecules30071531/s1; Scheme S1: The synthesis route of donor and acceptor; Figure S1: The overlap of the fluorescence spectra of donor and the absorption spectra of acceptor; Figure S2: The energy transfer in FRET process; Figure S3: The reaction of JXR under different pH conditions and solvent media (a) The fluorescence intensity ratio (I_470_/I_594_) of JXR relying on the variation in pH condition. (b) The fluorescence spectra of various pH. (c) The absorbance spectra of various pH. (d) The fluorescence spectra of JXR in DCM, 1,4-dioxane, THF, DMF, and DMSO. (e) The absorbance of JXR in DCM, 1,4-dioxane, THF, DMF, and DMSO.; Figure S4: The toxicity of JXR to RAW264.7 cells; Figure S5: The photostability of JXR in RAW264.7 cells. Figure S6: The HRMS of JXR; Figure S7: The ^1^H NMR of JXR; Figure S8: The ^13^C NMR of JXR; Figure S9: The ^1^H NMR of acceptor; Figure S10: The ^13^C NMR of acceptor. Table S1: Bifunctional probes based on FRET and TICT; Table S2: Bifunctional probes for ClO^−^ and viscosity. Refs. [62,63,64] are cited in the Supplementary Materials.
