# Supplementary material for: A Bifunctional Fluorescence Probe for the Detection of Hypochlorous Acid and Viscosity in Living Cells and Zebrafish
Source: Molecules. 2025 Mar 30;30(7):1531. doi: 10.3390/molecules30071531 (PMC11990390; doi:10.3390/molecules30071531)
Supplement: Supplementary file 1 [file molecules-30-01531-s001.zip › molecules-3506930-supplementary.pdf]

A Bifunctional Fluorescence Probe for the Detection of Hypochlorous Acid and Viscosity in Living Cells and Zebrafish

Xin Zhang [1,2,†], Yanmei Si [3,†], Xinpeng Chen [4], Xuqing Nie [2], Yiheng Zhang [2], Li Lin [1,2,*] and Yehao Yan [2,*]

1 School of Public Health, Shandong Second Medical University, Weifang 261053, China; zhangx_0928@163.com

2 School of Public Health, Jining Medical University, Jining 272067, China; n2796982732@163.com (X.N.); z19558669058@163.com (Y.Z.)

3 School of Forensic Medicine and Laboratory Medicine , Jining Medical University, Jining 272067, China; siyanmei90@126.com

4 School of Life Science, Hubei Normal University, Huangshi 435002, China; chenxinpeng@hbnu.edu.cn

* Correspondence: linli6711@sina.com (L.L.); yanyehao_322@163.com (Y.Y.)

† These authors contributed equally to this work.

Scheme. S1 The synthesis route of donor and acceptor.

Fig. S1 The overlap of the fluorescence emission spectra of donor and the absorption spectra of acceptor.

Fig. S2 The energy transfer in FRET process.

Fig. S3 The reaction of JXR under different pH conditions and solvent media.

Fig. S4 The toxicity of JXR to RAW 264.7 cells.

Fig. S5 The photo-stability of JXR in RAW 264.7 cells.

Fig. S6 The HRMS of JXR.

Fig. S7 The ^{1}H NMR of JXR.

Fig. S8 The ^{13}C NMR of JXR.

Fig. S9 The ^{1}H NMR of acceptor.

Fig. S10 The ^{13}C NMR of acceptor.

Table. S1 Bifunctional probes based on FRET and TICT

Table S2 Bifunctional probes for ClO^{-} and viscosity

1. Energy transfer efficiency

$E=1-F_{DA}/F_{D}$ (Equation S1)

Here, E is the energy transfer efficiency in FRET process. F_D is the fluorescent intensity of the donor. F_{DA} is the fluorescent intensity of the donor moiety in presence of acceptor [1].

2. Detection limit

$LOD = 3\boldsymbol{\sigma}/\mathbf{s}$ (Equation S2)

Herein, σ is the standard deviation of 10 groups JXR solution; s is the slope of linearity in fluorescence titration experiments [2].

3. Synthesis

3.1 The synthesis of precursors

The 7-(diethylamino)-2-oxo-2H-chromene-3-carboxylic acid (1 mmol, 261 mg) and 5-(4-hydroxypiperidin-1-yl)thiophene-2-carbaldehyde (1 mmol, 211 mg) were mixed in dichloromethane (10 mL) with the catalysis of N,N-dimethylpyridin-4-amine (DMAP, 0.4 mmol, 24.4 mg) and dicyclohexylmethanediimine (DCC, 3 mmol, 618 mg) [3]. After stirring for 36 h, the mixtures were separated by chromatographic column (silica gel: 200-300 mesh; DCM/MeOH V/V 90/1) with the yield of 19%。

The synthetic procedure was conducted as follows: 4-diethylaminosalicylaldehyde (386 mg, 0.02 mol) and diethyl malonate (64 mg, 0.04 mol) were dissolved in absolute ethanol (10 mL) with

piperidine (1.0 mL) as catalyst. The reaction mixture was refluxed with continuous stirring for 9 hours. Upon cooling to ambient temperature, the result-ing precipitate was isolated by vacuum filtration to yield the donor compound. The donor was synthesized according previous reported researches [4]. 1,4-dimethylpyridin-1-ium-iodide (1 mmol, 235 mg) and 5-(dimethylamino)thiophene-2-carbaldehyde (1 mmol, 155 mg) were dissolved in acetonitrile and refluxed for 12 h (**Scheme. S1**). Cooling the reaction solution down to room temperature, the crude product was filtered under reduced pressure. After recrystallization in acetonitrile solvent, pure product of acceptor was obtained with the yield of 23%. The structure of acceptor was demonstrated by ^{1}H NMR and ^{13}C NMR (**Fig. S9-10**). ^{1}H NMR (400 MHz, DMSO-d_6): δ = 3.08 (s, 6H), 4.08 (s, 3H), 6.08 (s, 1H), 6.46 (d, J = 16.0 Hz, 1H), 7.28 (s, 1H), 7.84 (d, J=8.0 Hz, 2H), 8.05 (d, J = 12.0 Hz, 1H) and 8.50 (d, J = 8 Hz, 2H). ^{13}C NMR (101 MHz, DMSO-d_6): 12.8, 22.7, 29.5, 44.2, 44.8, 46.5, 47.5, 68.8, 96.2, 106.1, 107.5, 107.6, 110.3, 114.6, 121.6, 125.1, 132.3, 136.0, 137.2, 144.3, 149.9, 153.4, 157.5 and 163.6.

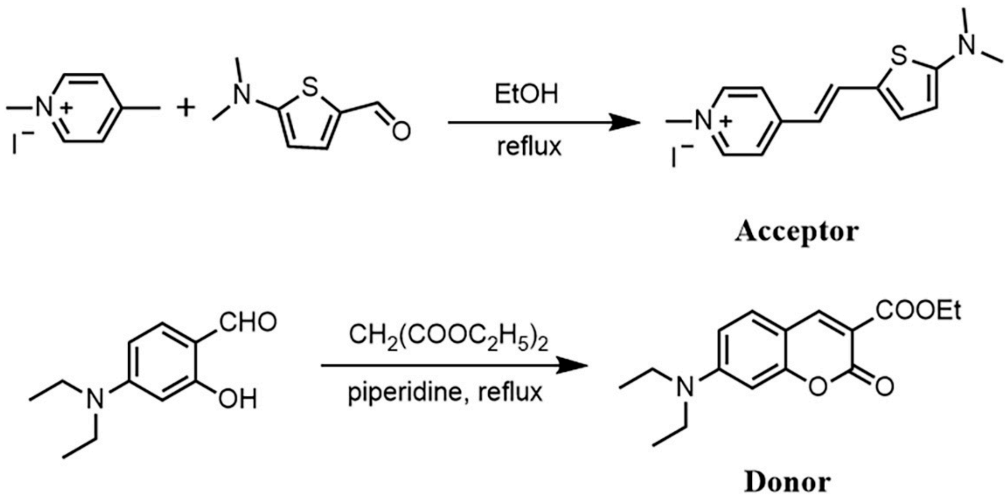

Scheme. S1 The synthesis route of acceptor.

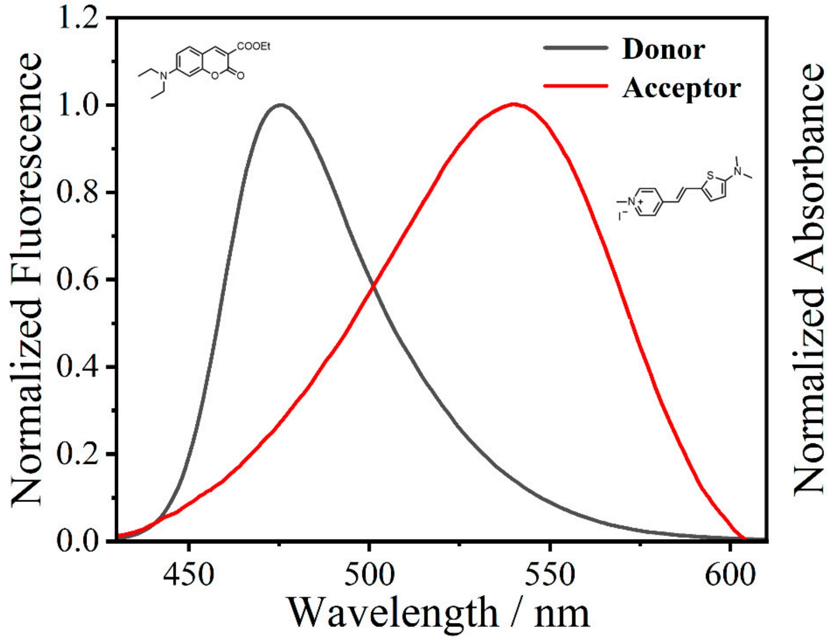

Fig. S1 The overlap of the normalized fluorescence spectra of donor (5 μM, ethanol) and the absorbance spectra of acceptor (5 μM, ethanol). (λ_{ex}: 400 nm; slit: 10/10)

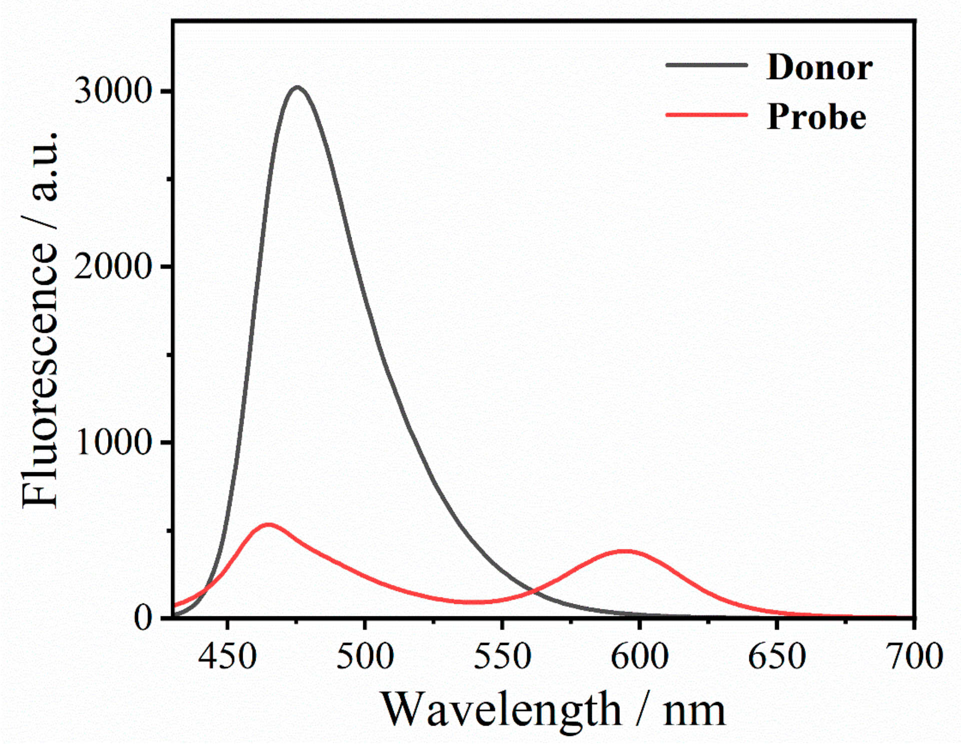

Figure S2 the fluorescence emission spectra of the donor (5 μM, black line) and probe JXR (5 μM, red line). (λ_{ex}: 400 nm; PBS; pH:7.4; slit: 10/10.)

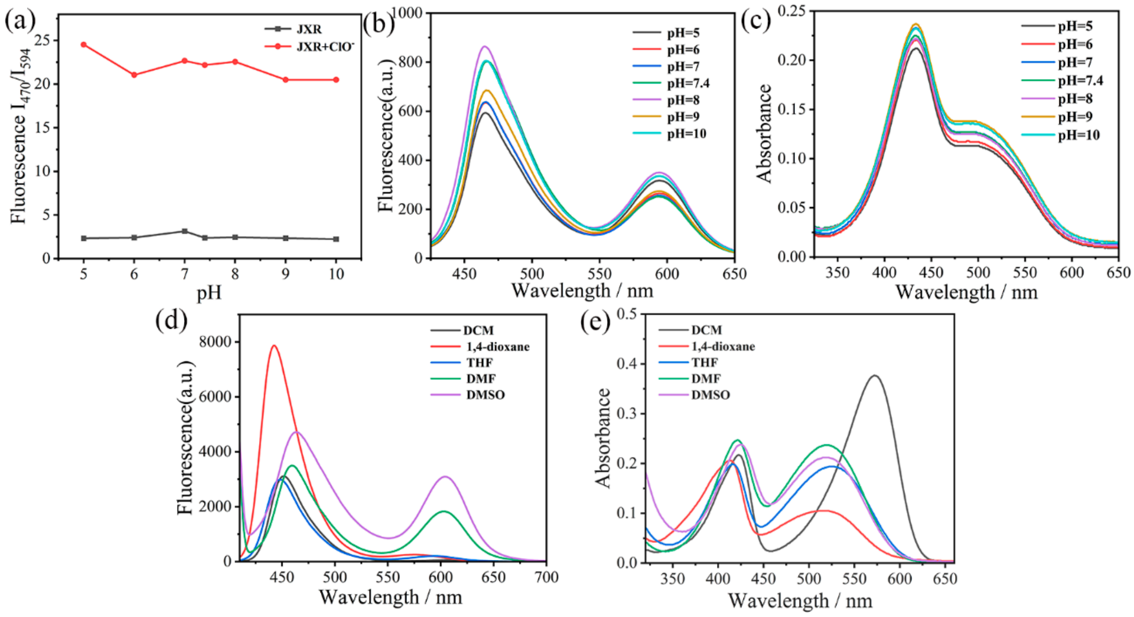

Figure S3 The reaction of JXR under different pH conditions and solvent media (a) The fluorescence intensity ratio (I_{470}/I_{594}) of JXR relying on the variation of pH condition. (b) The fluorescence spectra of various pH. (c) The absorbance spectra of

various pH. (d) The fluorescence spectra of JXR in DCM, 1,4-dioxane, THF, DMF and DMSO. (e) The absorbance of JXR in DCM, 1,4-dioxane, THF, DMF and DMSO. (5 μM, λ_{ex}: 400 nm; slit: 10/10)

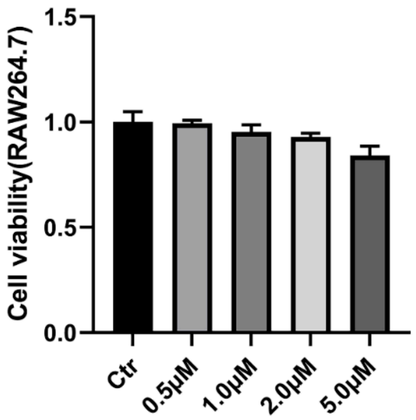

Figure S4 The toxicity of JXR to living cells. RAW264.7 cells were hatched for 2 h with 0, 0.5, 1.0, 2.0 and 5.0 μM JXR, respectively.

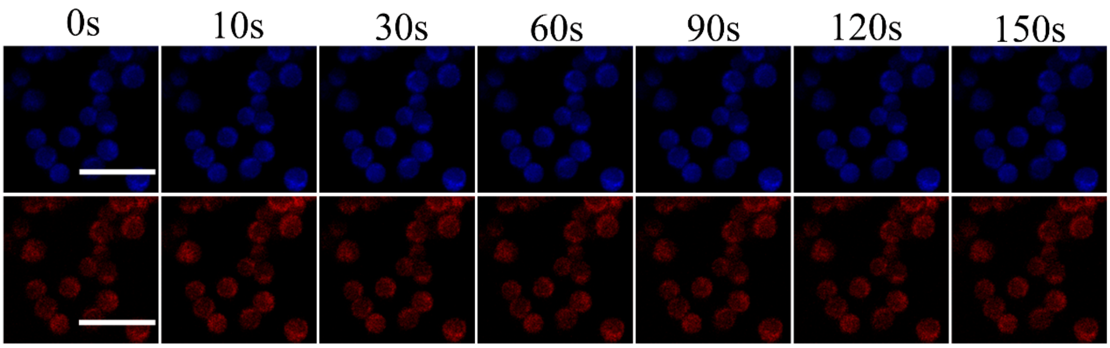

Figure S5 The confocal fluorescence imaging of living RAW264.7 cells cultured for 0.5 h with 2 mM JXR and irradiated for different time (0-150 s).

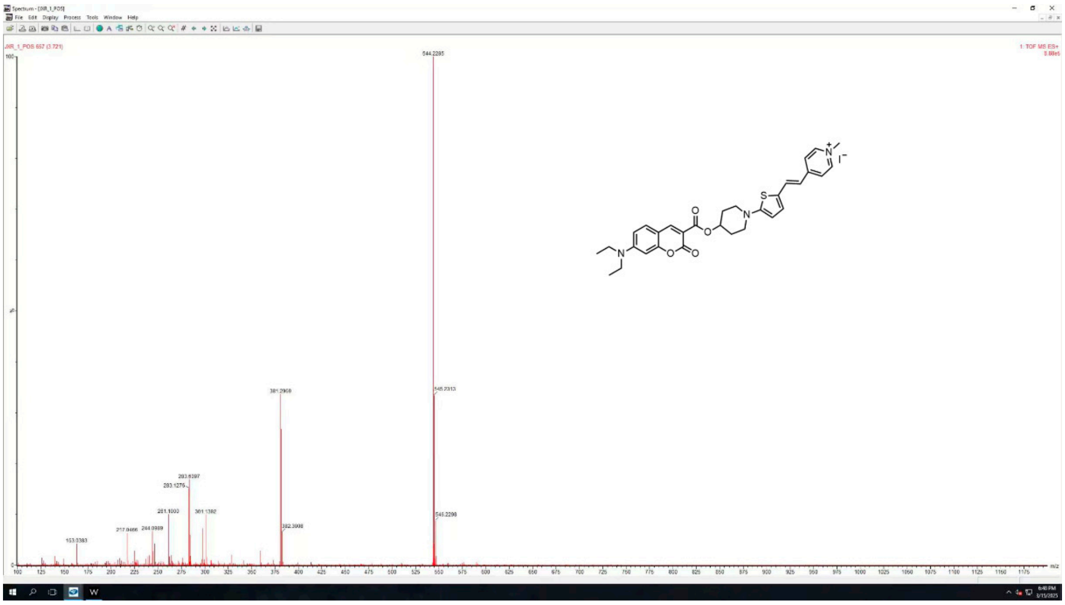

Figure S6 The HRMS of JXR.

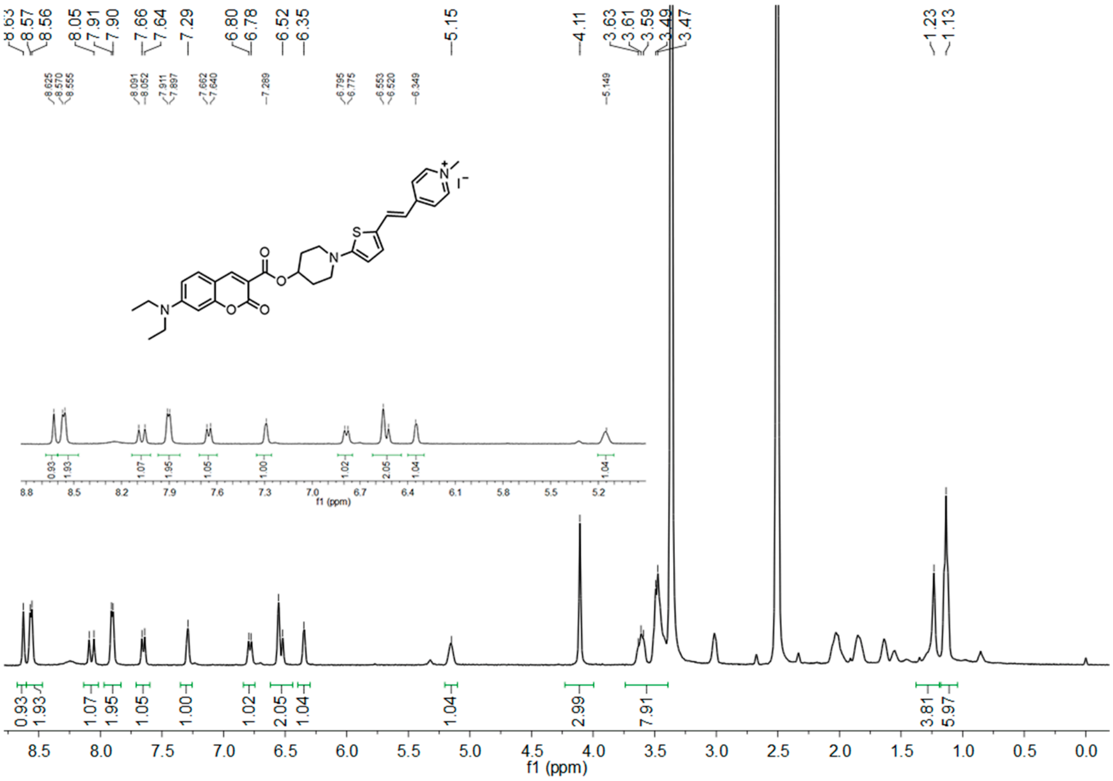

Figure S7 The ^{1}H NMR of probe JXR.

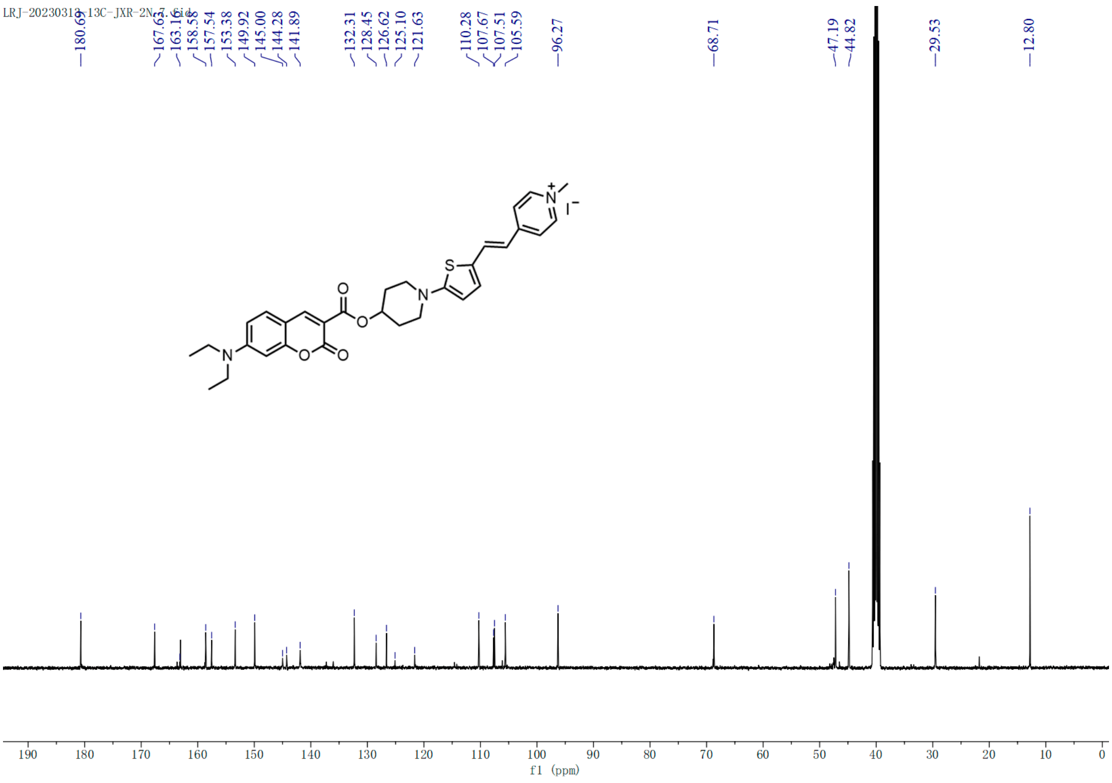

Figure S8 The ^{13}C NMR of probe JXR.

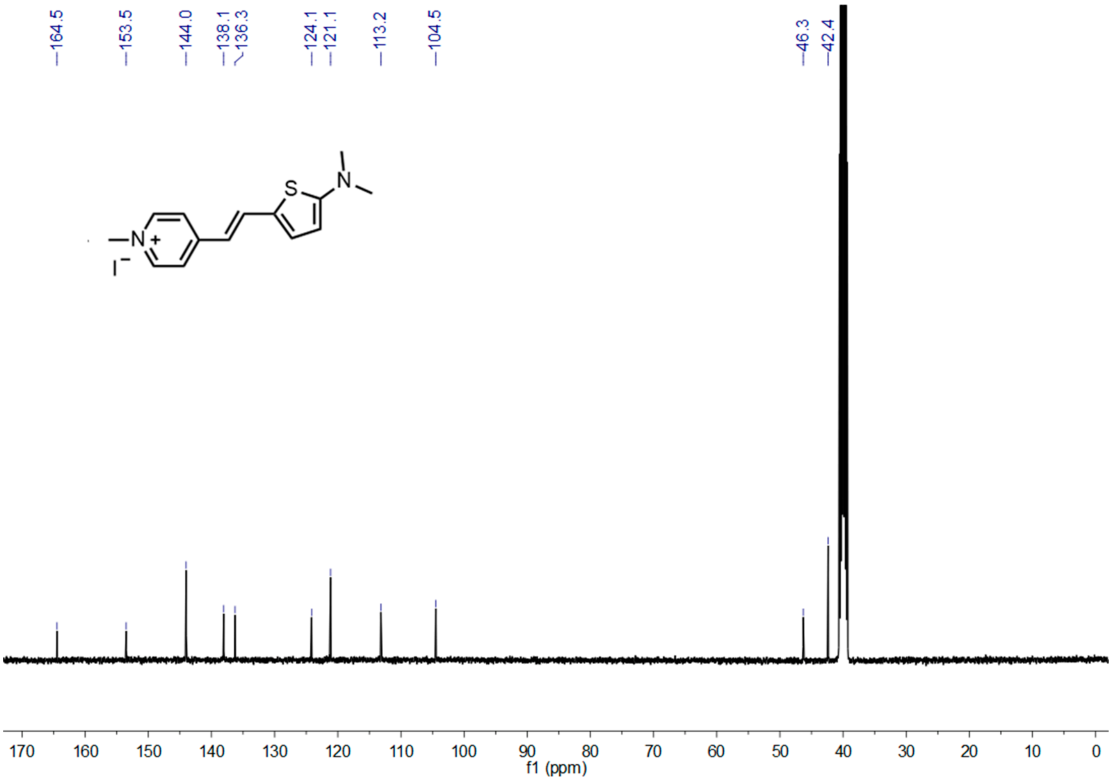

Figure S9 The ^{1}H NMR of acceptor.

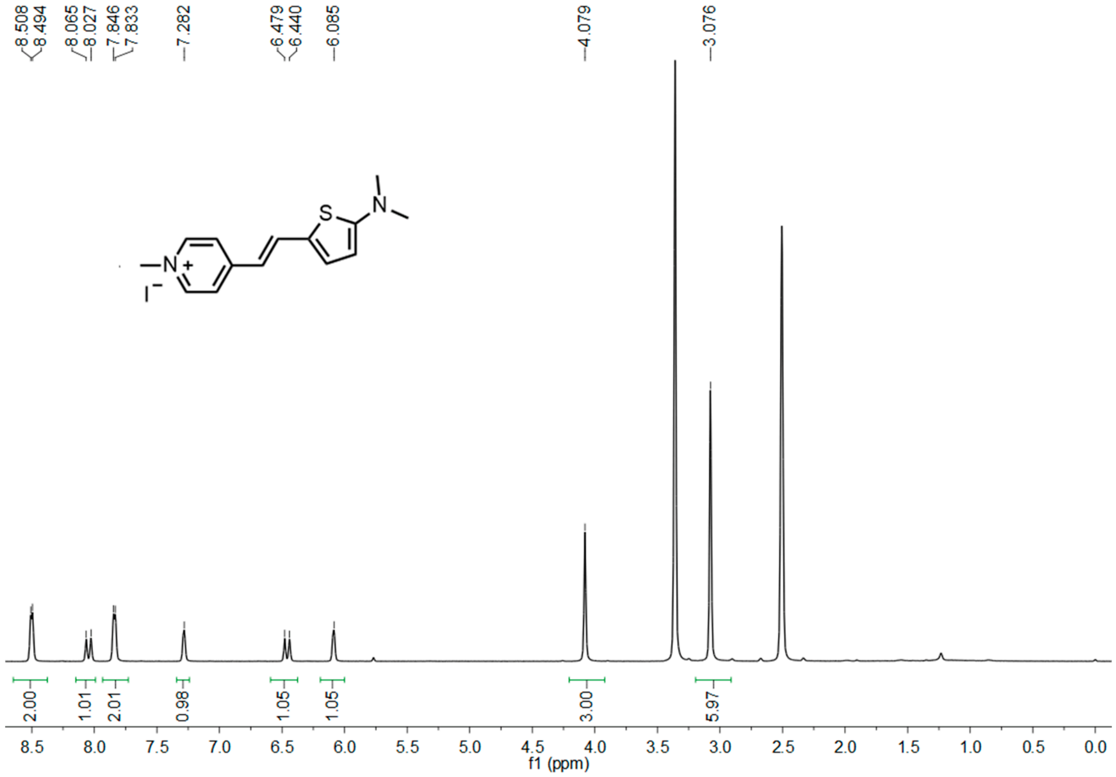

Figure S10 The ^{13}C NMR of acceptor.

Probe	Mechanism	test substance	application	Cell Targeting	Ref.
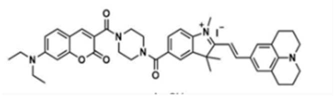	FRET/TICT	SO_2 / viscosity	living cell	No	*Talanta.* **2023**, 256, 124302
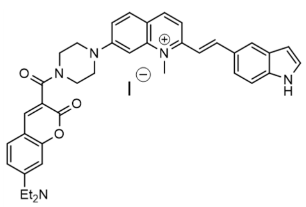	FRET/TICT	SO_2 / viscosity	food	Lysosomes	*Food Chem.* **2024**, 436, 137755
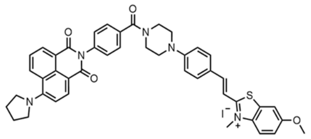	FRET/TICT	SO_2 / viscosity	living cell	Mitochondria	*Spectrochim. Acta, Part A.* **2025**, 325, 125074
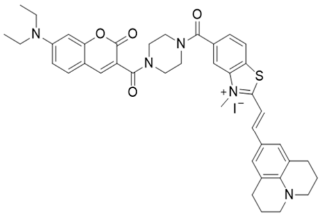	FRET/TICT	SO_2 / viscosity	living cell	Lysosomes	*Anal. Chim. Acta .* **2023**, 1239, 340721
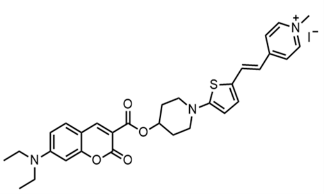	FRET/TICT	HClO / viscosity	living cell/ zebrafish	YES	This Work

Table S1 Bifunctional probes based on FRET and TICT

Probe' Structure	FRET	Detection Limits	Cell Target	Detection Medium	Application	Reference
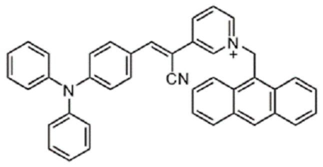	Yes	65.1nm	Mitochondria	PBS buffer with 30 % CH_3OH	Hela cells	*Talanta* **2022**, 241, 123235
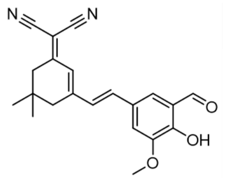	No	3.19mm	No	EtOH/H2O (3 : 7, v/v)	Hela cells Mice	*Org. Biomol. Chem.* **2023**,21, 1808-7812.
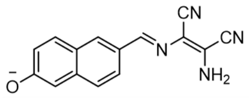	No	80.4nm	No	HEPES buffer with 30 % EtOH	A549 cells, Hela cells HepG2 cells, Diabetic Mice	*Sens. Actuators B Chem.* **2023**, 393, 134345 .
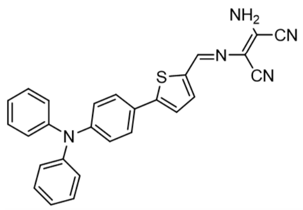	No	33nm	No	HEPES buffer with 30 % EtOH	Hela cells	*J. Mol. Liq.* **2024**,402 124788.
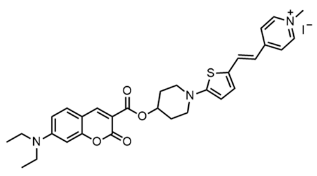	Yes	95.4nm	Mitochondria	PBS buffer	Raw264.7 cells Zebrafish	**This Work**

Table S2 Bifunctional probes for ClO^- and viscosity

Reference

[1] Liu, F.; Li, N.; Chen, Y.; Yu, H.; Miao, J.; Zhao, B. A quinoline-coumarin near-infrared ratiometric fluorescent probe for detection of sulfur dioxide derivatives. *Anal. Chim. Acta.* **2022**, 1211, 339908.

[2] Gao, C.; Chen, D.; Zhang, L.; Ma, M.; Liu, H.; Cui, H. A Mitochondria-Targeting Fluorescent Probe for the Dual Sensing of

Hypochlorite and Viscosity without Signal Crosstalk in Living Cells and Zebrafish. *Molecules.* **2024**, *29*, 3059.

[3] Seebacher, W.; Brun, R.; Kaiser, M.; Saf, R.; Weis, R. Synthesis and Evaluation of the Antitrypanosomal and Antiplasmodial Activities of New 4-Aminobicyclo[2.2.2]octane Derivatives. Eur. J. Med. Chem. 2005, 40 (9), 888–896.

[4] He,G.; Guo, D.; He, C.; Zhang, X.; Zhao, X.; Duan, C. A Color-Tunable Europium Complex Emitting Three Primary Colors and White Light, *Angew. Chem. Int. Ed.* **2009**, 48, 6132-6135.
